# Enhancing V2V Communication by Parsimoniously Leveraging V2N2V Path in Connected Vehicles

**DOI:** 10.3390/s26030819

**Published:** 2026-01-26

**Authors:** Songmu Heo, Yoo-Seung Song, Seungmo Kang, Hyogon Kim

**Affiliations:** 1 Department of Computer Science and Engineering, College of Informatics, Korea University, Anam-Dong, Sungbuk-gu, Seoul 02841, Republic of Korea; songmuh@korea.ac.kr (S.H.); salixkang@korea.ac.kr (S.K.); 2 Electronics and Telecommunication Research Institute, Gajeong-ro 218, Yuseong-gu, Daejeon 34129, Republic of Korea; yssong00@etri.re.kr

**Keywords:** V2V communication, V2N2V, connected vehicles, hybrid multipath, video streaming, QoE, cellular cost optimization

## Abstract

The rapid proliferation of connected vehicles equipped with both Vehicle-to-Vehicle (V2V) sidelink and cellular interfaces creates new opportunities for real-time vehicular applications, yet achieving ultra-reliable communication without prohibitive cellular costs remains challenging. This paper addresses reliable inter-vehicle video streaming for safety-critical applications such as See-Through for Passing and Obstructed View Assist, which require stringent Service Level Objectives (SLOs) of 50 ms latency with 99% reliability. Through measurements in Seoul urban environments, we characterize the complementary nature of V2V and Vehicle-to-Network-to-Vehicle (V2N2V) paths: V2V provides ultra-low latency (mean 2.99 ms) but imperfect reliability (95.77%), while V2N2V achieves perfect reliability but exhibits high latency variability ($P_{99}$: 120.33 ms in centralized routing) that violates target SLOs. We propose a hybrid framework that exploits V2V as the primary path while selectively retransmitting only lost packets via V2N2V. The key innovation is a dual loss detection mechanism combining gap-based and timeout-based triggers leveraging Real-Time Protocol (RTP) headers for both immediate response and comprehensive coverage. Trace-driven simulation demonstrates that the proposed framework achieves a 99.96% packet reception rate and 99.71% frame playback ratio, approaching lossless transmission while maintaining cellular utilization at only 5.54%, which is merely 0.84 percentage points above the V2V loss rate. This represents a 7× cost reduction versus PLR Switching (4.2 GB vs. 28 GB monthly) while reducing video stalls by 10×. These results demonstrate that packet-level selective redundancy enables cost-effective ultra-reliable V2X communication at scale.

## 1. Introduction

As the adoption of connected vehicles accelerates, vehicular communication technologies are reaching a critical inflection point. In 2024, the majority of passenger cars sold globally were equipped with embedded 4G/5G cellular connectivity as standard, and by 2028, over half of newly sold vehicles are expected to include 5G connectivity. In parallel, the standardization of Vehicle-to-Vehicle (V2V) direct communication has progressed steadily: Long-Term Evolution (LTE) Vehicle-to-Everything (V2X) was incorporated into 3GPP Release 14 (2017) and 5G-V2X into Release 16 (2020). Policy initiatives such as the U.S. National Highway Traffic Safety Administration (NHTSA)’s proposed V2V mandate in 2025 are expected to accelerate V2V deployment, resulting in vehicles equipped with both cellular-based V2N (Vehicle-to-Network) and direct-communication-based V2V interfaces [1].

This dual-interface architecture significantly expands the feasibility of various V2X applications requiring real-time data exchange between vehicles. The 5G Automotive Association (5GAA) defines multiple real-time video streaming use cases for inter-vehicle communication, as shown in Table 1. Applications such as See-Through for Passing, Obstructed View Assist, and Non-analyzed Sensor Signal Sharing commonly specify Service Level Objectives (SLOs) of less than 50 ms end-to-end latency with 99% reliability [2].

Inter-vehicle data sharing for these use cases can be realized through two fundamentally different paths. The first is direct communication via a V2V sidelink, and the second is network-relayed communication via cellular infrastructure, which we refer to as Vehicle-to-Network-to-Vehicle (V2N2V). These two paths exhibit contrasting characteristics. V2V direct communication provides ultra-low latency of a few milliseconds with minimal jitter through single-hop wireless links, but it suffers from fundamental reliability limitations due to wireless interference, signal blockage, and dynamic topology changes. In contrast, V2N2V communication achieves high reliability through managed cellular infrastructure, but it incurs relatively higher end-to-end latency and latency variability under network load [3], and it may impose an economic burden for the sustained exchange of large-volume sensor data over extended periods, depending on the selected data plan.

For the V2N2V path, achieving the 50 ms/99% SLO presents fundamental challenges in conventional cellular deployments. Research by Coll-Perales et al. [3] reports that in centralized multi-Mobile Network Operator (MNO) environments, the 90th percentile latency reaches approximately 58 ms, which is attributed to delays induced by Remote Peering Points for inter-operator traffic exchange and latency variability in the public Internet segment. Our measurements from actual urban driving environments confirm these findings: while the mean latency of 39.40 ms appears acceptable, the $P_{90}$ reaches 59.86 ms and $P_{99}$ reaches 120.33 ms, which is more than double the SLO threshold. This latency is fundamentally incompatible with ultra-reliable real-time V2X applications.

**Figure 1:**
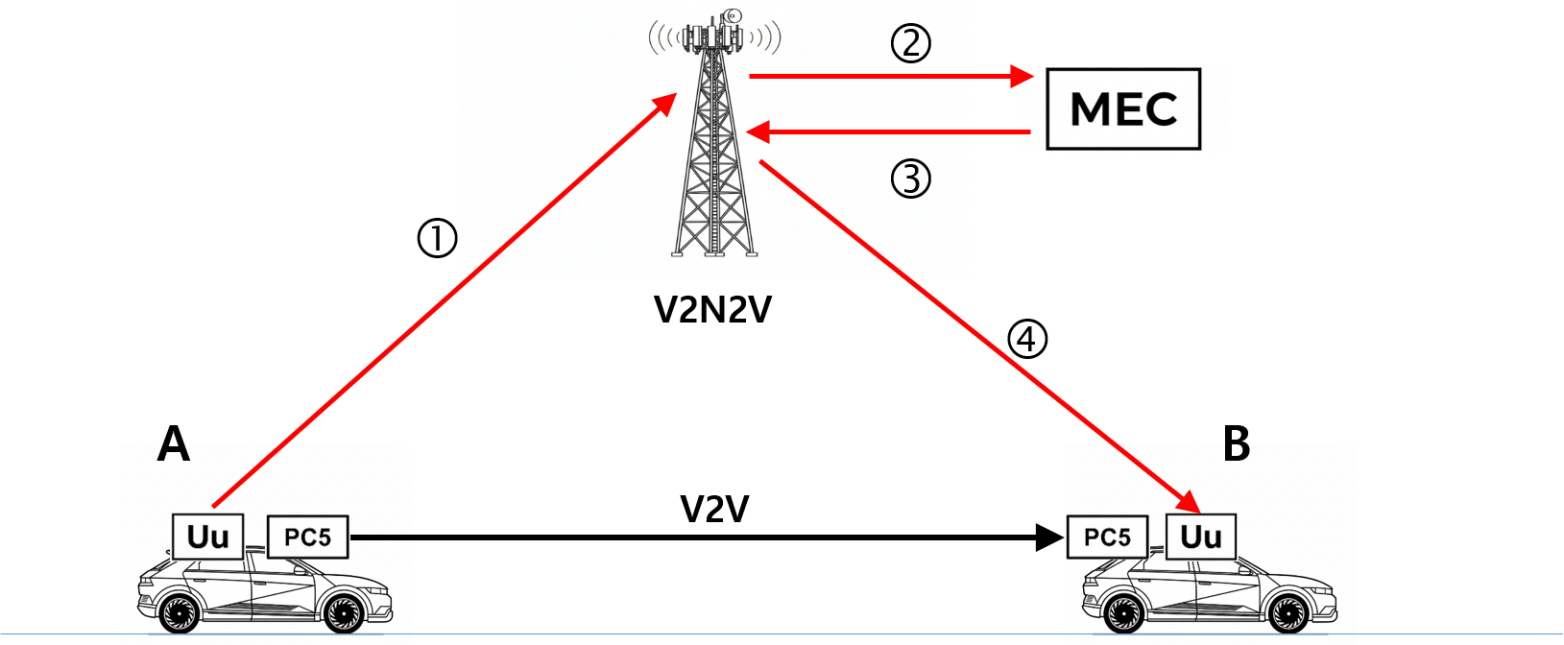
Communication paths from A to B: V2V vs. V2N2V.

To address this limitation, 3GPP standardized Mobile Edge Computing (MEC) architectures specifically for Ultra-Reliable Low-Latency Communication (URLLC) applications in Release 17 [4]. MEC@gNB deployment with local User Plane Function (UPF) breakout reduces end-to-end latency by eliminating remote routing: traffic terminates at the base station edge rather than traversing the core network and Internet. Coll-Perales et al. [3] demonstrate that MEC@gNB reduces $P_{90}$ latency from 58 ms to 3.6 ms, making the 50 ms/99% SLO achievable. Consequently, this paper assumes MEC-based edge architecture as the target deployment environment and proposes a hybrid data-sharing strategy that exploits the complementary characteristics of V2V and V2N2V in this architecture (Figure 1).

The core idea of this paper is a selective redundancy strategy that utilizes V2V as the low-latency, cost-free primary path while performing selective retransmission via V2N2V only when packet loss occurs on V2V. Unlike existing mode-switching approaches that incur high costs by shifting all traffic to cellular upon V2V link quality degradation, the proposed framework achieves both cost efficiency and reliability by recovering only lost packets through V2N2V.

The main contributions of this paper are as follows:

- Measurement-based path characterization: Through real-world urban driving experiments, we quantify the complementary nature of V2V (low latency, imperfect reliability) and V2N2V (high reliability, variable latency) paths.
- Hybrid framework design: We introduce an on-demand retransmission strategy that utilizes V2N2V as a backup path only upon packet loss on the V2V link, simultaneously achieving data reliability and minimizing cellular overhead.
- Performance validation: Through trace-driven simulation, we demonstrate that the proposed framework achieves 99.96% packet reception rate and 99.71% frame playback success rate while providing approximately 7× cellular cost reduction compared to existing approaches [5].

## 2. Related Work

Recent studies highlight the fundamental role of V2X communication in establishing robust Intelligent Transportation Systems (ITSs). Comprehensive surveys and taxonomies demonstrate how V2X technologies are evolving to enhance traffic efficiency and safety through interconnected vehicular frameworks [6,7]. In parallel, the adoption of Mobile Edge Computing (MEC) is becoming increasingly critical. Research into future vehicular networks, including 6G and edge-based streaming optimization, validates the necessity of edge-assisted architectures to support bandwidth-intensive and intelligent applications [8,9]. Against this backdrop of evolving infrastructure, ensuring reliable real-time transmission remains a primary challenge. Autonomous and connected vehicles require diverse V2X applications with varying QoS requirements, often demanding high bandwidth, high reliability, and ultra-low latency simultaneously. These multifaceted requirements are difficult to satisfy with a single Radio Access Technology (RAT), motivating extensive research on multi-RAT vehicular networks. Below, we categorize existing approaches by their use of cellular infrastructure.

### 2.1. Multi-RAT V2V Without Cellular Infrastructure

Sepulcre and Gozalvez [10] proposed a context-aware algorithm where vehicles exchange Channel Busy Ratio (CBR) information and select the RAT with the lowest load among those satisfying reliability requirements, with hysteresis to prevent frequent switching. Jacob et al. [11] demonstrated that redundant transmission over IEEE 802.11p [12] and LTE-V2X PC5 simultaneously can significantly extend the communication range and reception success rate even under high congestion. Yacheur et al. [13,14] applied Deep Reinforcement Learning to determine the optimal transmission mode (single, redundant, or split) per message in ITS-G5/LTE-PC5 environments.

However, these approaches rely solely on V2V direct paths, which have inherent reliability limitations under challenging propagation conditions, motivating our hybrid approach utilizing V2N2V as a supplementary backup.

### 2.2. Cellular-Supported Multi-RAT Without Cost Consideration

Jacob et al. [15] proposed a Hybrid Communications Management layer for DSRC and LTE-V2X (PC5 and Uu), defining communication profiles as the selection unit and enabling adaptive multiprofile transmission for throughput or reliability enhancement. Albonda et al. [16] proposed dynamic selection between Cellular (Uu) and Sidelink (PC5) based on SINR and resource availability at the cluster level with spatial resource reuse between distant clusters. González et al. [17] presented a Multi-RAT Dual Connectivity architecture where vehicles connect to both LTE and NR base stations, using PC5 and Uu independently or in parallel for reliability enhancement through packet duplication.

### 2.3. Cellular-Supported Multi-RAT with Cost Consideration

Since commercial cellular networks incur usage-based costs, several studies explicitly minimize cellular infrastructure usage.

Brahim et al. [5] activated cellular only when the DSRC packet loss exceeds a threshold with minimum retention time and hysteresis for stable switching. Hui et al. [18] modeled inter-vehicle cooperation as a Coalition Formation Game, deriving Nash-stable access strategies that minimize download costs while suppressing unnecessary cellular usage. Mir et al. [19,20] proposed QoS-aware RAT selection that first adjusts the beacon rate during DSRC congestion and utilizes LTE only when the QoS remains unsatisfied. Altahrawi et al. [21] combined LSTM-based RSSI prediction with an analytic hierarchy process for service-oriented RAT selection optimizing efficiency and cost. Mir et al. [22] proposed traffic steering maintaining DSRC as the default and offloading to LTE only during congestion, combining uplink unicast and downlink eMBMS for cellular efficiency. Khalid et al. [23] proposed a decision tree-based selection of optimal technology and transmission frequency based on context (distance, latency, speed, traffic density). Chowdhury et al. [24] prioritized WiFi and DSRC-based multi-hop V2V cooperation, restricting cellular to minimum representative vehicles for CDN-based live streaming. Bréhon-Grataloup et al. [25] combined location-based QoS grids with PC5 relay for connection survivability, minimizing cellular fallback in urban NLOS environments.

Table 2 summarizes these multi-RAT multipath approaches.

## 3. Problem Description

This section examines the characteristics of V2N2V paths that connected vehicles can provide compared to V2V paths. Understanding the characteristics of these two paths, which will be used in combination, is essential for finding reliable and economically rational solutions for V2V applications, which is our goal. This will also provide an objective perspective on the various multipath combination approaches we reviewed in the previous section.

### 3.1. Characteristics of V2V vs. V2N2V Paths

V2V and cellular network communication (V2N2V) paths exhibit distinct latency and reliability characteristics. To quantitatively characterize V2V and V2N2V paths, we conducted measurement experiments for each path during actual driving.

#### 3.1.1. Experimental Setup

Driving experiments were conducted in Seoul’s inner city, which represents typical urban driving environments.

##### Equipment and Configuration

**Figure 2:**
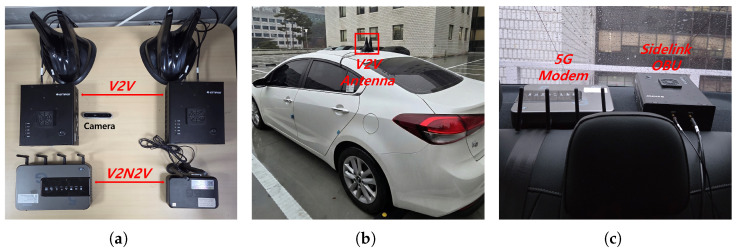
Experimental setup for real-world driving experiments. (**a**) Devices configuration: antennas, PC5 modems, and Uu modems; (**b**) V2V antenna installation; (**c**) In-vehicle devices installation.

The two vehicles participating in the experiments were equipped with vehicular communication devices. Figure 2 shows the configuration and placement of this equipment. Each vehicle had a 5G modem for Uu interface and a sidelink on-board unit (OBU) for the PC5 interface, respectively. They were connected to a laptop for data processing along with a front-facing camera. All communication equipment was placed inside the cabin except the V2V antenna that was mounted on the roof.

- V2V Link Setup: For the V2V communication measurement, we used Ettifos’ Software-Defined Radio (SDR)-based 5G NR OBU. Table 3 summarizes the NR Sidelink Mode 2 configuration used in our experiments. We employed a non-periodic resource allocation scheme by setting the Resource Reservation Period (RRP) to zero, which results in dynamic resource selection for each transmission. This configuration is well suited for aperiodic video traffic, as it allows the transmitter to flexibly select resources in response to irregular frame arrivals unlike semi-persistent resource reservations with fixed periodicity. Furthermore, in the current real-world driving environment, neighboring vehicles rarely perform concurrent V2X transmissions, making resource collisions negligible. Therefore, while more advanced resource allocation and coordination mechanisms will be necessary in future high-density scenarios, the adopted configuration is sufficient for evaluating the intrinsic performance characteristics of the V2V link.
- V2N2V Path Setup: For V2N2V communication measurement, we employed 5G modems from two different mobile operators (Korea Telecom and LGU+). In commercial cellular network environments, direct Internet Protocol (IP) communication between terminals is restricted due to Network Address Translation (NAT) structures where terminals (i.e., 5G modems) are given private IP addresses. Accordingly, to enable end-to-end communication between the two 5G modems, we configured a communication path via a Traversal Using Relays around NAT (TURN) server on the Internet.

##### Video Traffic Coding and Transmission

In the measurements, real-time video with a resolution of 640×480 at 30 frames per second (fps) was encoded using the H.264 codec, implemented via FFmpeg, and transmitted via the Real-time Transport Protocol (RTP). To satisfy real-time constraints, the encoder was configured to disable B-frames, set the Group of Pictures (GoP) size to 30, and enable zero-latency mode.

Since NR Sidelink resource allocation (Mode 2) has fixed periodicity, transmitting frame-level video traffic as is cannot be naturally accommodated by the Sidelink. It is because a single video frame has to be transmitted in multiple RTP packets due to the relatively small sidelink resource sizes. Consequently, an instantaneous packet burst occurs upon every video frame arrival. To cope with this issue, we set the resource period to 1 ms (aligned with the 1 ms slot structure in Numerology μ=0) and applied packet pacing that limited the RTP packet transmission interval to 1 ms. This approach is essential for stably loading traffic onto the sidelink channel despite variable video frame sizes. Additionally, Hybrid Automatic Repeat Request (HARQ) functionality was enabled in sidelink transmission, performing up to three retransmissions excluding the initial transmission.

#### 3.1.2. V2V Path: Low Latency and Imperfect Reliability

V2V communication achieves low latency through direct transmission. Our urban driving experiments show that while the V2V path delivers ultra-low latency with minimal jitter, it falls short of the reliability requirements for our target applications.

##### Low Latency

**Figure 3:**
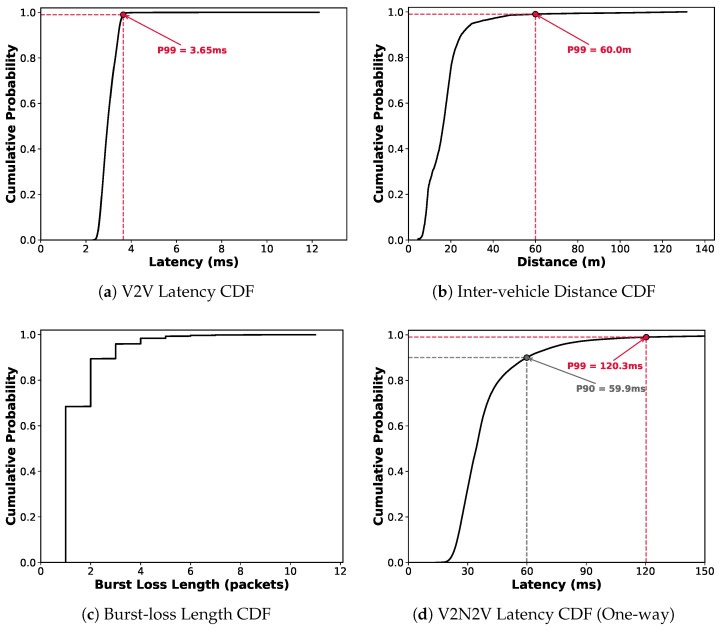
Measured characteristics of communication paths: (**a**) V2V latency, (**b**) inter-vehicle distance, (**c**) V2V burst loss length, and (**d**) V2N2V latency.

The cumulative distribution function (CDF) in Figure 3a visually demonstrates the excellent latency characteristics of the V2V path. Despite using SDR equipment, the mean latency was only 2.99 ms, and the 99th percentile ($P_{99}$) was merely 3.65 ms. The standard deviation representing latency variability (jitter) was also very low at 0.32 ms, showing stable latency performance. The maximum latency of 12.30 ms observed in the tail region is attributed to HARQ retransmission operations to recover transient errors.

##### Reliability Limitations

In contrast, data reliability revealed problems. As shown in Figure 3b, the two vehicles maintained close proximity, ensuring conducive wireless channel conditions, with an average distance of 17.19 m (standard deviation 10.99 m) throughout the experiment. Despite this highly favorable communication environment where the 99th percentile ($P_{99}$) remained within 59.98 m, the Packet Reception Ratio (PRR) was only 95.77%. This residual loss occurred despite the HARQ retransmission mechanism operating up to three times, suggesting that V2V links alone cannot satisfy the ultra-reliability required by target services. The experimental results demonstrate that V2V communication reliability must be conservatively estimated depending on environmental factors such as complex urban structures and vehicle blocking.

#### 3.1.3. V2N2V Path: High Reliability and Large, Variable Latency

The V2N2V path available to connected vehicles has high reliability through the managed cellular infrastructure. However, the longer path results in higher latency compared to V2V, and latency jitter is also inevitably high due to dynamic resource scheduling influenced by variable cell user counts and loads.

##### High Reliability

In inter-vehicle video transmission experiments using 5G modems, a total of 243,597 RTP packets transmitted during approximately 19 min of driving achieved a 100% reception rate without a single loss. This demonstrates that commercial 5G cellular networks can provide high reliability even for video data communication.

##### Latency Limitations

Despite high reliability, the V2N2V path exhibits clear latency limitations. As shown in Figure 3d, the mean latency of 39.40 ms appears to satisfy the SLO (50 ms). However, the tail distribution reveals a different picture: the 90th percentile ($P_{90}$) reaches 59.86 ms, and the 99th percentile ($P_{99}$) reaches 120.33 ms. This indicates that achieving our target requirement, latency within 50 ms with 99% reliability is structurally infeasible. The $P_{99}$ exceeds the threshold by 2.4 times, and even $P_{90}$ already surpasses 50 ms. In other words, more than 10% of packets experience latency spikes beyond the allowable limit, demonstrating that V2N2V as is cannot guarantee ultra-low latency.

This finding aligns with prior research. Coll-Perales et al. [3] report that the tail latency induced by jitter in 5G end-to-end communication is substantial, making it practically difficult to ensure that more than 99% of packets arrive within 50 ms, especially in multi-MNO environments or centralized cloud architectures. Our measured $P_{99}$ latency of 120.33 ms confirms this limitation. The experimental setup, where 5G modems from different operators (Korea Telecom, LGU+) communicated via a TURN server on the public Internet, represents a typical multi-MNO deployment without edge computing support. In such centralized architectures, inter-vehicle packets traverse a lengthy path: vehicle A → gNB → core network → Internet peering point → core network → gNB → vehicle B, accumulating substantial queuing and propagation delays. Therefore, in this paper, we assume an MEC-based architecture where the application server is deployed at the network edge, significantly reducing the V2N2V path latency to complement V2V reliability limitations. Table 4 shows that the MEC@gNB architecture can achieve the latency reduction necessary for ultra-reliable V2X applications.

Fortunately, MEC deployment at gNB with local UPF breakout, standardized in 3GPP Release 17 [4], eliminates this lengthy routing path. Therefore, for performance evaluation in Section 5, we will adopt the MEC@gNB latency model from Coll-Perales et al. [3] as our target deployment architecture. Specifically, we utilize the multi-MNO scenario provided by this model, which incorporates local peering latency, to ensure a realistic evaluation of inter-operator connectivity.

It is important to note that in our framework, the MEC node serves solely as a low-latency relay point for packet forwarding rather than a computation platform; thus, edge computing power is not a critical factor. Consequently, we posit MEC coverage as a structural prerequisite for the V2N2V path, as standard cellular routing cannot meet the strict latency constraints of the target application.

### 3.2. Implications of V2V-Only Transmission on QoE

By analyzing the V2V trace, this section considers the QoE implication of using the V2V path alone without the hybrid V2V+V2N2V framework proposed in this paper. V2V packet loss does not directly translate to QoE degradation in real-time video transmission. Structured codecs like H.264 exhibit packet-to-frame dependency, inter-frame dependency, and error propagation, which combine to amplify single packet losses nonlinearly.

#### 3.2.1. Packet-to-Frame Dependency

A single video frame encoded with H.264 is fragmented into multiple RTP packets for transmission. Since all packets constituting a frame must be received for successful decoding, even a single packet loss causes decoding failure of the entire frame. In our V2V measurement trace, the 4.23% packet loss rate was amplified to 8.83% frame loss rate: only 41,708 out of 45,464 video frames could be decoded.

#### 3.2.2. Inter-Frame Dependency

H.264’s inter-frame prediction achieves high compression efficiency but introduces structural vulnerability to data loss. When an I-frame or preceding P-frame is lost, subsequent P-frames referencing them become undecodable even if their own packets arrive intact. Of the 41,708 decodable frames, only 38,745 were playable due to reference dependencies, yielding a frame loss rate of 14.78%, which is 3.5 times the original packet loss rate. During the 26-minute experiment, 1522 playback stalls occurred, averaging one interruption per second.

#### 3.2.3. Error Propagation

Even when decoding succeeds, error propagation causes continuous quality degradation. Regions restored through error concealment propagate residual errors to subsequent frames via H.264’s motion compensation, causing artifacts to persist until a new I-frame refreshes the Group of Pictures (GoP). The V2V-only path recorded an average SSIM of 0.8625, which is classified as poor quality [26].

### 3.3. Summary

Using V2V alone, the 4.23% packet loss rate was amplified to 14.78% frame loss with 1522 playback stalls and an average SSIM of 0.8625. We need to resolve this problem for the targeted applications. After the proposed solution approach to the problem is presented in Section 4, the V2V-only results will serve as the baseline for evaluation in Section 5.3.

## 4. Solution Approach

This section proposes a practical strategy that utilizes both V2V and V2N2V paths to address the problems raised in the previous section while reflecting the characteristics of both paths observed in Section 3.1 and minimizing monetary costs by limiting the use of the paid V2N2V path to essential cases only.

### 4.1. V2V Data Transmission Augmented with V2N2V Retransmission

The core idea of the proposed framework is to use V2V as the main data path and parsimoniously use the V2N2V path for only retransmission requests and reply on the packet level. Since the framework operates at the application layer, it is agnostic to the underlying RAT, aligning with the ITS station architecture [27], which is designed to accommodate heterogeneous access technologies (e.g., IEEE 802.11p for V2V and 3GPP Uu for V2N).

#### 4.1.1. Overall Workflow

**Figure 4:**
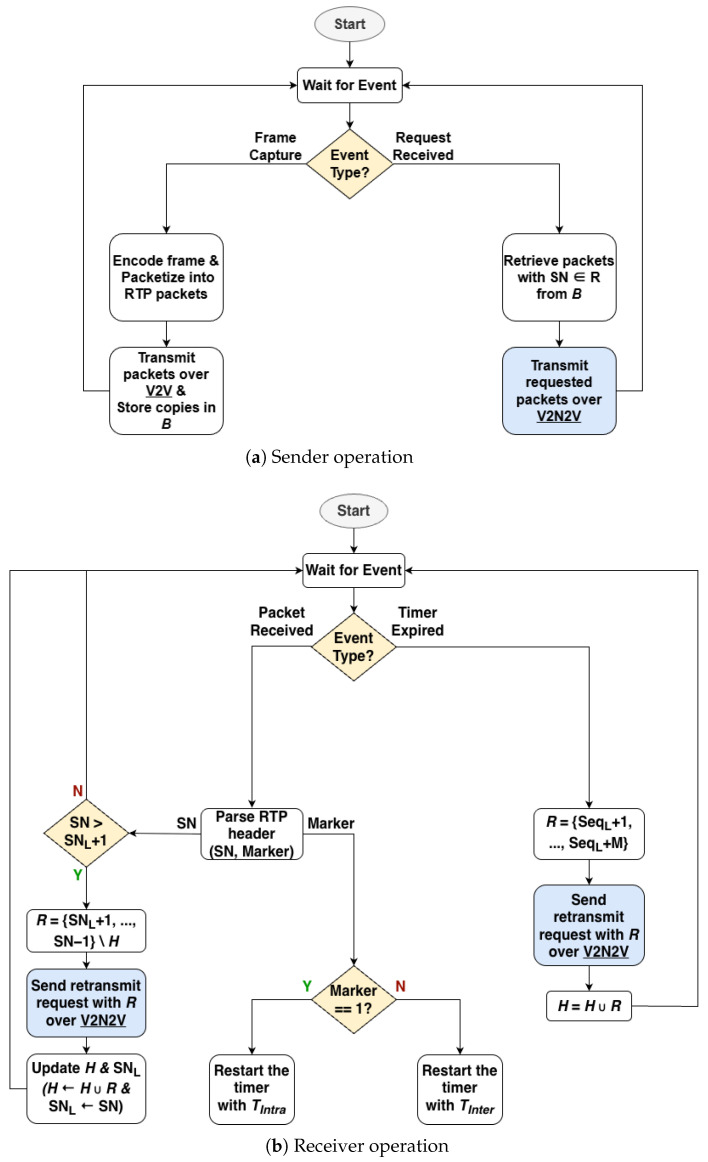
Flow chart of the proposed algorithm; *B*: packet buffer at sender side, *R*: set of sequence numbers to be retransmitted, *H*: set of sequence numbers of packets already requested for retransmission, $SN_{L}$: last successfully received sequence number, $T_{intra}$: intra-frame retransmission timeout, $T_{inter}$: inter-frame retransmission timeout.

The detailed operational procedure of the proposed framework is described in Figure 4. The algorithm consists of dual-path management at the sender side and loss detection and request logic at the receiver side.

- At Sender: When a video frame is generated, the sender terminal encodes it, packetizes it into RTP packets, transmits via V2V, and simultaneously stores in local buffer (*B*) for retransmission handling. When retransmission requests (*R*) are received via V2N2V, only the corresponding packets are transmitted via the V2N2V path by referencing the buffer.
- At Receiver: The receiver terminal operates based on packet reception events and timeout events. When a packet is received, the RTP sequence number is examined to verify continuity with the previously received packet. When a gap in sequence numbers is detected, the missing sequence numbers are determined as lost packets and added to set *R*, and retransmission is immediately requested via the V2N2V path. Simultaneously, the RTP Marker bit of the received packet is checked to identify frame boundaries, and retransmission timers are set according to the Marker value. When the Marker is 0, the $T_{Intra}$ timer is set to respond to intra-frame packet loss; when the Marker is 1, the $T_{Inter}$ timer is set for inter-frame loss detection with each timer being reset and restarted accordingly. When timeout occurs, packets that have not yet arrived after the last successfully received sequence number are considered lost and added to *R*, and retransmission is requested.

#### 4.1.2. Loss Detection Mechanisms

**Figure 5:**
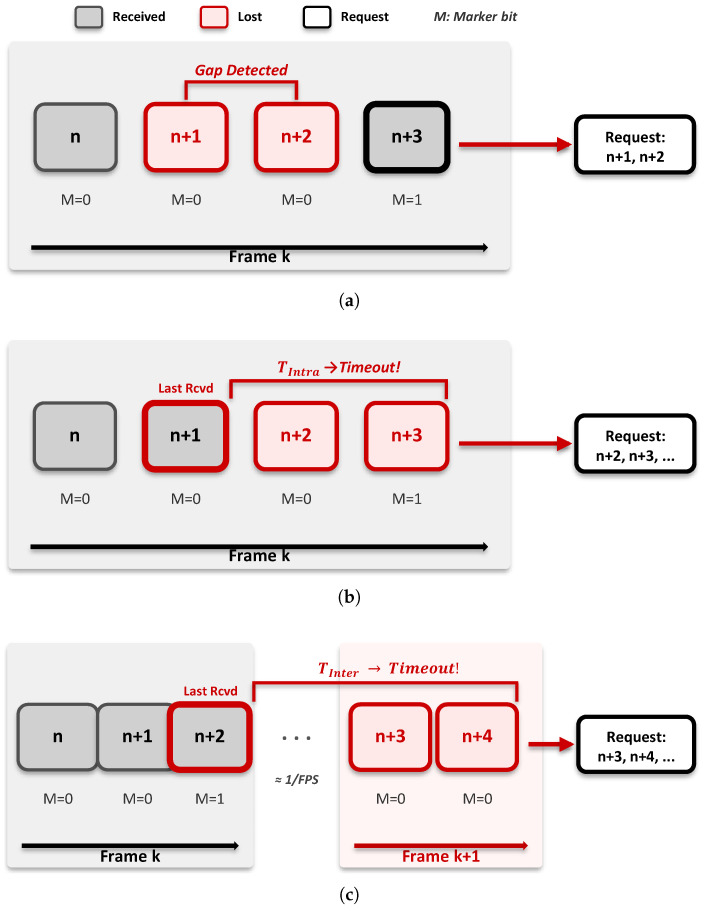
Loss detection mechanisms: (**a**) gap-based trigger, (**b**) intra-frame timeout, and (**c**) inter-frame timeout.

This framework applies two complementary trigger mechanisms utilizing RTP header information for rapid and accurate packet loss detection. They are illustrated in Figure 5.

##### Gap-Based Trigger

The most immediate loss detection method is examining the RTP sequence number continuity. When a gap (k>1) occurs between the previously received packet (seq=n) and the current packet (seq=n+k), the receiver determines packets seq=n+1,…,n+k−1 as lost and immediately requests retransmission. Indeed, our 26-minute V2V trace (173,673 packets) confirmed zero out-of-order arrivals, with 100% of sequence numbers arriving in monotonically increasing order with occasional losses. This is expected in the V2V sidelink due to single-hop transmission without intermediate routing nodes and MAC-layer HARQ preserving the original sequence order. Even if sporadic reordering were to occur, a consequent false gap detection and retransmission request would only cause a duplicate packet delivery, which would be safely discarded. Figure 5a shows an example.

##### Timeout-Based Trigger

Since gap-based detection requires subsequent packet arrivals, it cannot identify loss when no packets arrive for some duration. To address this, we use parallel timeout triggers with values differentiated by the RTP Marker bit, which indicates the last packet of a video frame.

- Intra-frame Timeout ($T_{Intra}$): When the Marker bit is 0, this indicates that constituent packets of the currently receiving frame still remain. Since the transmission interval between packets within a frame is very short (1 ms), a short timeout ($T_{Intra}$) is applied to respond quickly to intra-frame loss. Figure 5b shows an example of this case.
- Inter-frame Timeout ($T_{Inter}$): When the Marker bit is 1, this indicates that the transmission of the frame is complete. Since the next packet will not arrive until the next video frame is generated, a relatively long timeout ($T_{Inter}$) considering the video frame rate is applied to prevent unnecessary retransmission requests. That is, $T_{Inter}$ expires only when it can be determined that the first packet of the next frame has not arrived. Figure 5c shows an example of this case.

#### 4.1.3. Request Optimizations

To minimize unnecessary cellular usage, we apply two optimizations:

- The receiver maintains a request history set (*H*) to filter duplicate requests. Packets already in *H* do not generate additional requests even if detected as lost again by subsequent triggers.
- Since the exact length of the loss interval cannot be known in advance in timeout-based retransmission requests, packets after $SN_{L}$ that were not actually lost may be requested. To mitigate this problem, the number of retransmitted packets is limited in timeout-based retransmission requests.

#### 4.1.4. Other System Details

The sender maintains a packet buffer *B* for selective retransmission. Given the 50 ms SLO and 1 ms pacing interval, at most 50 packets (≈75 KB) need to be buffered. Packets are evicted once they exceed the SLO window. At the receiver, packets whose end-to-end network latency exceeds 50 ms are regarded as invalid and are discarded by comparing the packet transmission timestamp with the final reception time. When duplicate packets are received, the receiver consistently uses the first-arriving packet and discards subsequent copies.

Each retransmission request consists of IP/UDP headers (28 bytes) and a payload specifying the starting RTP sequence number and count. Gap-based requests may include an optional exclusion field for packets already requested; timeout-based requests always specify contiguous ranges. The request overhead is negligible: for instance, over a 26-min experiment, gap-based detection generated 307.69 KB of request traffic (0.16% of video data), while the gap-and-timeout triggers generated 334.12 KB (0.18%).

## 5. Performance Evaluation

This section develops an analytical model for the proposed hybrid V2V+V2N2V recovery scheme, validates it through trace-driven simulation, and analyzes system performance comprehensively.

### 5.1. Analytical Framework

#### 5.1.1. System Model

We consider a video stream encoded using H.264/AVC with a Group of Pictures (GoP) structure. Each GoP consists of one I-frame followed by (G−1) P-frames. The I-frame serves as a reference and can be decoded independently, while each P-frame depends on all preceding frames in the GoP for successful decoding. This dependency structure implies that if frame *k* fails to decode, all subsequent frames k+1,k+2,…,G−1 in the same GoP become unplayable. Let $N_{f}^{\left(I\right)}$ and $N_{f}^{\left(P\right)}$ denote the average number of packets in an I-frame and P-frame, respectively. Packets are transmitted with a pacing interval of δ ms.

Table 5 lists the baseline parameter values used throughout the analysis.

#### 5.1.2. Channel Error Model

Vehicular wireless channels exhibit bursty error patterns due to multipath fading, shadowing, and interference. Under an independent and identically distributed (IID) packet loss model with loss probability *p*, the frame failure probability would be

(1) $$P_{f}^{\mathrm{IID}}=1-{\left(1-p\right)}^{N_{f}}$$

However, vehicular channels typically exhibit significantly more bursty behavior than the IID model predicts. When losses are bursty, multiple packet losses concentrate within the same frame rather than spreading across different frames. This concentration effect reduces the number of frames that experience failure compared to IID losses. To capture this burstiness, we introduce a burstiness factor β<1:

(2) $$P_{f}\left(N_{f}\right)=1-{\left(1-p\right)}^{\beta \cdot N_{f}}$$

Other channel parameters are directly measurable from the V2V trace:

- Packet loss rate *p* is determined from the measured PRR:
  (3) $$p=1-{\mathrm{PRR}}_{V2V}^{\mathrm{trace}}=1-0.9577=0.0423$$
- The burst termination rate *q* is estimated from the burst length CDF in Figure 3c, which shows that 95.92% of bursts contain three or fewer packets. Assuming a geometric distribution for burst length, we obtain q=0.656, corresponding to an expected burst length of E[m]=1/q=1.52 packets.

#### 5.1.3. V2N2V Backup Path Model

We model the V2N2V round-trip time as a shifted log-normal distribution:

(4) $$T_{V2N2V}=d_{\min}+X,\;X∼\mathrm{LogNormal}\left(\mu ,\sigma^{2}\right)$$

The probability that V2N2V recovery completes within budget *B* is

(5) $$P_{V2N2V}\left(B\right)=\Phi \left(\frac{\ln(B-d_{\min})-\mu }{\sigma }\right),\;B>d_{\min}$$

where Φ(·) is the standard normal CDF.

#### 5.1.4. Frame Playback Rate Analysis

##### V2V-Only Baseline

In the V2V-only scheme, a frame is successfully decoded only if all its packets arrive. The frame decode probabilities are

(6) $$\begin{array}{cc} P_{d}^{\left(I\right)} & ={\left(1-p\right)}^{\beta \cdot N_{f}^{\left(I\right)}} \end{array}$$

(7) $$\begin{array}{cc} P_{d}^{\left(P\right)} & ={\left(1-p\right)}^{\beta \cdot N_{f}^{\left(P\right)}} \end{array}$$

Due to the GoP dependency structure, frame *k* is playable only if all preceding frames are decoded. The frame playback rate is

(8) $$\mathrm{FPR}=\frac{P_{d}^{\left(I\right)}}{G}\sum_{k=0}^{G-1}{\left(P_{d}^{\left(P\right)}\right)}^{k}=\frac{P_{d}^{\left(I\right)}}{G}\cdot \frac{1-{\left(P_{d}^{\left(P\right)}\right)}^{G}}{1-P_{d}^{\left(P\right)}}$$

##### Proposed Scheme

The proposed scheme adds V2N2V recovery when packet loss is detected on the V2V path. A frame is successfully decoded if either (a) no packet loss occurs, or (b) all lost packets are successfully recovered via V2N2V within the SLO deadline.

Detection Mechanism Interaction: A critical design constraint arises from the interaction between loss detection mechanisms. With intra-frame timeout $T_{\mathrm{intra}}=2.5$ ms and packet pacing interval δ=1 ms, the detection trigger depends on burst length *m*:

- m≤2: The next packet arrives within m·δ≤2 ms $<T_{\mathrm{intra}}$, triggering gap-based detection. All *m* lost packets are identified and requested.
- m≥3: The timeout fires at $T_{\mathrm{intra}}=2.5$ ms before the next packet arrives at m·δ≥3 ms, triggering timeout-based detection. Only N=3 packets are requested.

This threshold $m^{*}=\left\lfloor T_{\mathrm{intra}}/\delta \right\rfloor =2$ determines whether full or partial recovery is possible.

Burst Length Distribution: Based on the analysis of the real-life V2V trace, we assume that the burst length follows a geometric distribution with termination probability q=0.656:

(9) $$P\left(m=k\right)={\left(1-q\right)}^{k-1}q,\;k=1,2,3,\ldots$$

Key probabilities:

(10) $$\begin{array}{ccc} P(m\leq 2) & = & 1-{\left(1-q\right)}^{2}=0.8817\;(\mathrm{gap}-\mathrm{based}\;\mathrm{detection}) \end{array}$$

(11) $$\begin{array}{ccc} P(m\geq 3) & = & {\left(1-q\right)}^{2}=0.1183\;(\mathrm{timeout}-\mathrm{based}\;\mathrm{detection}) \end{array}$$

(12) $$\begin{array}{ccc} P(m>3|m\geq 3) & = & \frac{{\left(1-q\right)}^{3}}{{\left(1-q\right)}^{2}}=1-q=0.344\;\left(\mathrm{unrecoverable}\right) \end{array}$$

Recovery Analysis by Scenario: We analyze recovery success based on the burst length and position within the frame.

Scenario 1: Gap-based detection (m≤2 and $s+m<N_{f}$). When burst length m≤2 and the burst does not extend to the frame boundary ($s+m<N_{f}$), the next packet (index s+m) arrives within m·δ≤2 ms, triggering gap-based detection before $T_{\mathrm{intra}}=2.5$ ms expires. The recovery budget is

(13) $$B_{\mathrm{gap}}=T_{\mathrm{SLO}}-\left(s+m\right)\cdot \delta$$

For I-frames ($N_{f}^{\left(I\right)}\approx 20$), the worst case s+m=19 yields $B_{\mathrm{gap}}=31$ ms. For P-frames ($N_{f}^{\left(P\right)}\approx 3$), the worst case yields $B_{\mathrm{gap}}=48$ ms. Both provide sufficient time:

(14) $$P_{\mathrm{rec}}^{\mathrm{gap}}=P_{V2N2V}\left(B_{\mathrm{gap}}\right)\approx 1$$

Scenario 2: Intra-frame timeout (m≥3, mid-frame). When m≥3 and the burst occurs mid-frame (s>0, $s+m<N_{f}$), $T_{\mathrm{intra}}$ triggers after packet s−1. Only N=3 packets are requested. The recovery budget is

(15) $$B_{\mathrm{intra}}=T_{\mathrm{SLO}}+\delta -T_{\mathrm{intra}}$$

Recovery succeeds only if both conditions hold: (a) V2N2V completes within budget, and (b) burst length m≤N:

(16) $$P_{\mathrm{rec}}^{\mathrm{intra}}=P_{V2N2V}\left(B_{\mathrm{intra}}\right)\cdot P\left(m\leq N|m\geq 3\right)=P_{V2N2V}\left(B_{\mathrm{intra}}\right)\cdot q$$

With q=0.656, only 65.6% of timeout-triggered bursts are recoverable.

Scenario 3: Tail loss ($s+m\geq N_{f}$, s>0). When the burst extends to the frame boundary, $T_{\mathrm{intra}}$ triggers. The tail loss size is $L_{\mathrm{tail}}=N_{f}-s$. Recovery requires $L_{\mathrm{tail}}\leq N$:

(17) $$P_{\mathrm{rec}}^{\mathrm{tail}}\left(s\right)=P_{V2N2V}\left(B_{\mathrm{intra}}\right)\cdot 1\left[N_{f}-s\leq N\right]$$

Only positions where $s\geq N_{f}-N$ allow recovery.

Scenario 4: Frame-start burst (s=0). When the burst begins at the first packet, inter-frame timeout $T_{\mathrm{inter}}$ triggers after the previous frame completes. The detection delay is

(18) $$\tau_{\mathrm{inter}}=\left(N_{f}^{\mathrm{prev}}-1\right)\cdot \delta +T_{\mathrm{inter}}-T_{\mathrm{frame}}$$

The recovery budget becomes

(19) $$B_{\mathrm{timeout}}=T_{\mathrm{SLO}}-\tau_{\mathrm{inter}}$$

For frames following an I-frame ($N_{f}^{\mathrm{prev}}=N_{f}^{\left(I\right)}=20.41$),

(20) $$\tau_{\mathrm{inter}}^{\left(I\right)}=19.41+60-33.33=46.08\;\mathrm{ms},\;B_{\mathrm{timeout}}^{\left(I\right)}=3.92\;\mathrm{ms}$$

For frames following a P-frame ($N_{f}^{\mathrm{prev}}=N_{f}^{\left(P\right)}=2.69$),

(21) $$\tau_{\mathrm{inter}}^{\left(P\right)}=1.69+60-33.33=28.36\;\mathrm{ms},\;B_{\mathrm{timeout}}^{\left(P\right)}=21.64\;\mathrm{ms}$$

Recovery requires both V2N2V completion and m≤N:

(22) $$P_{\mathrm{rec}}^{\mathrm{timeout}}=P_{V2N2V}\left(B_{\mathrm{timeout}}\right)\cdot P\left(m\leq N\right)$$

Combined Recovery Probability: Let $P_{\mathrm{gap}}=P\left(m\leq m^{*}\right)=0.8817$ denote the probability of gap-based detection. For a frame of size $N_{f}$, the overall recovery probability combines all scenarios weighted by their occurrence probabilities.

For mid-frame bursts (s>0):

(23) $$P_{\mathrm{rec}}^{\mathrm{mid}}=P_{\mathrm{gap}}\cdot P_{\mathrm{rec}}^{\mathrm{gap}}+\left(1-P_{\mathrm{gap}}\right)\cdot P_{\mathrm{rec}}^{\mathrm{intra}}$$

Substituting $P_{\mathrm{rec}}^{\mathrm{gap}}\approx 1$ and $P_{\mathrm{rec}}^{\mathrm{intra}}=P_{V2N2V}\left(B_{\mathrm{intra}}\right)\cdot q$,

(24) $$P_{\mathrm{rec}}^{\mathrm{mid}}\approx P_{\mathrm{gap}}+\left(1-P_{\mathrm{gap}}\right)\cdot P_{V2N2V}\left(B_{\mathrm{intra}}\right)\cdot q$$

With baseline parameters yielding $P_{V2N2V}\left(B_{\mathrm{intra}}\right)\approx 1$:

(25) $$P_{\mathrm{rec}}^{\mathrm{mid}}\approx 0.8817+0.1183\times 0.656\approx 0.9593$$

The overall recovery probability for a frame averages over burst start positions:

(26) $$P_{\mathrm{rec}}\left(N_{f}\right)=\frac{1}{N_{f}}\cdot P_{\mathrm{rec}}^{\mathrm{timeout}}+\frac{N_{f}-1}{N_{f}}\cdot P_{\mathrm{rec}}^{\mathrm{mid}}$$

Frame Playback Rate: The frame decode probability with recovery is

(27) $$P_{d}^{\mathrm{prop}}\left(N_{f}\right)=\left(1-P_{f}\left(N_{f}\right)\right)+P_{f}\left(N_{f}\right)\cdot P_{\mathrm{rec}}\left(N_{f}\right)$$

where $P_{f}\left(N_{f}\right)=1-{\left(1-p\right)}^{\beta \cdot N_{f}}$ is the frame failure probability.

For I-frames and P-frames:

(28) $$\begin{array}{cc} P_{d}^{\left(I\right)} & =\left(1-P_{f}^{\left(I\right)}\right)+P_{f}^{\left(I\right)}\cdot P_{\mathrm{rec}}^{\left(I\right)} \end{array}$$

(29) $$\begin{array}{cc} P_{d}^{\left(P\right)} & =\left(1-P_{f}^{\left(P\right)}\right)+P_{f}^{\left(P\right)}\cdot P_{\mathrm{rec}}^{\left(P\right)} \end{array}$$

Then, Equation (8) can be used to compute the FPR for the proposed scheme.

Dominant Failure Modes The analysis identifies two primary failure modes:

1. Long bursts (m>N): When m>3 and timeout-based detection triggers, only three packets are recovered. The remaining m−3 packets are lost, causing frame failure. This occurs with probability $P\left(m>3\right)={\left(1-q\right)}^{3}=4.08\%$ of all bursts.
2. Tight timeout budget after I-frames: The recovery budget $B_{\mathrm{timeout}}^{\left(I\right)}=3.92$ ms is marginal for a V2N2V round-trip, reducing the recovery success probability for frame-start bursts following I-frames.

#### 5.1.5. Sensitivity Analysis

**Figure 6:**
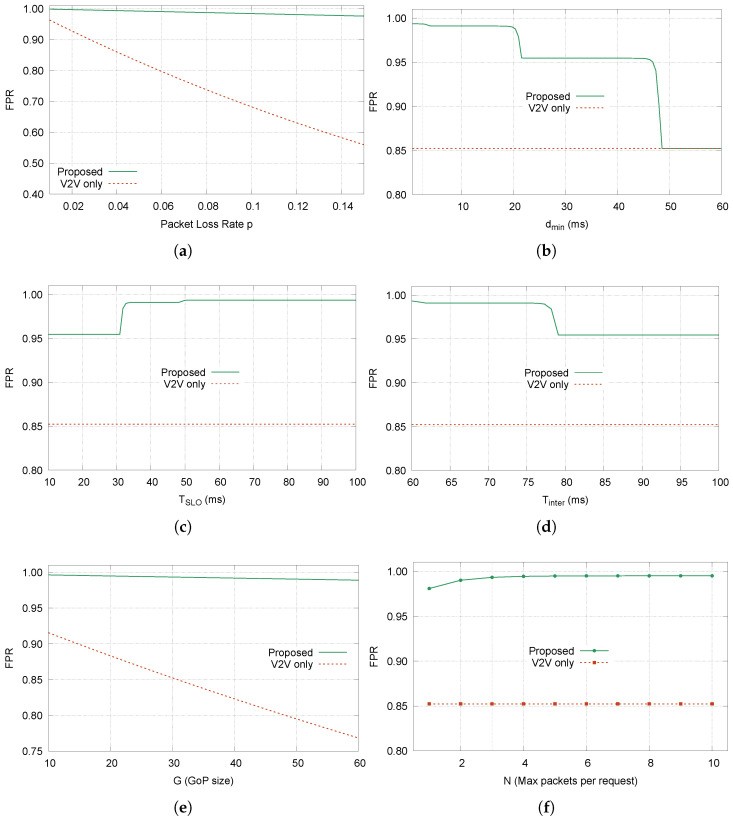
FPR sensitivity to system parameters; red dashed: V2V-only baseline. Green solid: proposed scheme. (**a**) FPR vs. *p*; (**b**) FPR vs. $d_{min}$; (**c**) FPR vs. $T_{SLO}$; (**d**) FPR vs. $T_{inter}$; (**e**) FPR vs. *G*; (**f**) FPR vs. *N*.

We analyze how FPR varies with key parameters. Figure 6 shows the results, where β is set to the fitted value determined in Section 5.4.

##### Packet Loss Rate (*p*)

The packet loss rate has the strongest impact on V2V-only performance. As *p* increases from 0.01 to 0.15, the V2V-only FPR drops from 0.95 to below 0.55. In contrast, the proposed scheme maintains FPR above 0.97 across the entire range. This demonstrates the robustness of V2N2V backup: even when the direct V2V path experiences significant degradation, the cellular backup path successfully recovers most lost frames.

##### Minimum V2N2V Delay ($d_{\min}$)

The FPR curve for $d_{\min}$ exhibits three distinct drop regions, each corresponding to a critical recovery budget threshold. To understand this behavior, we first identify the three recovery budgets in the system:

- $B_{\mathrm{timeout}}^{\left(I\right)}=T_{\mathrm{SLO}}-\tau_{\mathrm{inter}}^{\left(I\right)}=3.92$ ms: budget for frame-start bursts following an I-frame;
- $B_{\mathrm{timeout}}^{\left(P\right)}=T_{\mathrm{SLO}}-\tau_{\mathrm{inter}}^{\left(P\right)}=21.64$ ms: budget for frame-start bursts following a P-frame;
- $B_{\mathrm{intra}}=T_{\mathrm{SLO}}+\delta -T_{\mathrm{intra}}=48.5$ ms: budget for mid-frame bursts.

Recovery via V2N2V is possible only when $d_{\min}<B$, since the minimum round-trip time cannot exceed the available budget. As $d_{\min}$ increases, it sequentially exceeds each threshold:

First drop ($d_{\min}\approx 4$ ms). When $d_{\min}$ exceeds $B_{\mathrm{timeout}}^{\left(I\right)}=3.92$ ms, frame-start burst recovery becomes impossible for frames following an I-frame. This is the tightest constraint because I-frames are large ($N_{f}^{\left(I\right)}=20.41$ packets), causing the inter-frame timeout to trigger late:

(30) $$\tau_{\mathrm{inter}}^{\left(I\right)}=\left(N_{f}^{\left(I\right)}-1\right)\cdot \delta +T_{\mathrm{inter}}-T_{\mathrm{frame}}=46.08\;\mathrm{ms}$$

This leaves only $T_{\mathrm{SLO}}-46.08=3.92$ ms for recovery. The FPR drop at this point is relatively small because frame-start bursts are infrequent (probability $1/N_{f}$ for each frame).

Second drop ($d_{\min}\approx 22$ ms). When $d_{\min}$ exceeds $B_{\mathrm{timeout}}^{\left(P\right)}=21.64$ ms, frame-start burst recovery also fails for frames following P-frames. Since P-frames are smaller ($N_{f}^{\left(P\right)}=2.69$ packets), $\tau_{\mathrm{inter}}^{\left(P\right)}=28.36$ ms provides more recovery time. Beyond this threshold, all frame-start bursts become unrecoverable.

Third drop ($d_{\min}\approx 48.5$ ms). When $d_{\min}$ exceeds $B_{\mathrm{intra}}=48.5$ ms, even mid-frame burst recovery fails. Since mid-frame bursts constitute the majority of loss events (probability $\left(N_{f}-1\right)/N_{f}$), this causes a dramatic FPR collapse to the V2V-only level (≈0.85).

The multi-step degradation pattern reveals the relation between the network latency and application timing constraints with I-frame-related recovery being the most vulnerable due to the large I-frame size.

##### Latency Deadline ($T_{\mathrm{SLO}}$)

The FPR curve for $T_{\mathrm{SLO}}$ exhibits a distinctive three-stage staircase pattern as the deadline increases. This behavior arises because each recovery budget becomes viable (i.e., exceeds $d_{\min}$) at a different $T_{\mathrm{SLO}}$ threshold. Beyond $T_{\mathrm{SLO}}\approx 50$ ms, all three recovery budgets comfortably exceed $d_{\min}$, and $P_{V2N2V}\left(B\right)\approx 1$ for all scenarios. The FPR curve saturates at approximately 0.993, which is limited only by the residual probability of

- Bursts longer than N=3 packets (undetectable within the request window);
- V2N2V delays exceeding the available budget (tail of the log-normal distribution).

Design implications. The staircase pattern reveals that $T_{\mathrm{SLO}}$ requirements are driven primarily by the I-frame bottleneck. The baseline value of $T_{\mathrm{SLO}}=50$ ms is positioned just above the third threshold (48.6 ms), ensuring all recovery mechanisms are active with minimal margin.

For applications requiring lower latency, the analysis suggests that

- $T_{\mathrm{SLO}}\geq 31$ ms is sufficient for most recovery scenarios (Stages 1–2), achieving FPR ≈0.95;
- $T_{\mathrm{SLO}}\geq 49$ ms is necessary for full recovery capability (all three stages), achieving FPR ≈0.99.

##### Inter-Frame Timeout ($T_{\mathrm{inter}}$)

The inter-frame timeout is designed to detect frame-start losses: cases where the first packet of a new frame is lost, leaving no gap-based detection opportunity. However, even without any loss, there is a natural delay between the last packet of frame *k* and the first packet of frame k+1:

(31) $$\Delta t_{\mathrm{inter}}=T_{\mathrm{frame}}+\epsilon_{\mathrm{enc}}+\epsilon_{\mathrm{jitter}}$$

where $T_{\mathrm{frame}}=33.33$ ms is the frame interval, $\epsilon_{\mathrm{enc}}$ is the encoder processing variability, and $\epsilon_{\mathrm{jitter}}$ is the network jitter. Our V2V trace measurements show that the 99th percentile of inter-frame packet arrival intervals is 59.08 ms. If $T_{\mathrm{inter}}$ is set below this value, the timeout will trigger even when no loss has occurred, causing false-positive retransmission requests.

In Figure 6d, the sharp transition at $T_{\mathrm{inter}}\approx 77$–80 ms occurs when $B_{\mathrm{timeout}}^{\left(P\right)}$ approaches $d_{\min}=2.5$ ms. At $T_{\mathrm{inter}}=80$ ms, $B_{\mathrm{timeout}}^{\left(P\right)}=1.64$ ms $<d_{\min}$, making frame-start burst recovery impossible for all frame types. Beyond 80 ms, FPR stabilizes at a reduced level because only mid-frame recovery (with budget $B_{\mathrm{intra}}=48.5$ ms, unaffected by $T_{\mathrm{inter}}$) remains functional. Beyond 80 ms, FPR stabilizes at a reduced level because only mid-frame recovery remains functional. The system does not collapse to V2V-only performance because $B_{\mathrm{intra}}$ provides sufficient margin for the majority of burst events.

##### GoP Size (*G*)

The FPR curves for *G* reveal a contrast: while V2V-only FPR degrades significantly as *G* increases, the proposed scheme maintains nearly constant performance. In Equation (8), the summation $\sum_{k=0}^{G-1}{\left(P_{d}^{\left(P\right)}\right)}^{k}$ represents the expected number of consecutive decodable P-frames before a failure. Its behavior depends critically on how close $P_{d}^{\left(P\right)}$ is to unity:

- If $P_{d}^{\left(P\right)}<1$: The sum converges to $1/(1-P_{d}^{\left(P\right)})$ as G→∞, making FPR ∝1/G;
- If $P_{d}^{\left(P\right)}\approx 1$: The sum ≈G, making FPR $\approx P_{d}^{\left(I\right)}$ (independent of *G*).

With V2N2V recovery, the P-frame decode probability reaches $P_{d}^{\left(P\right)}=0.9997$. At this level, the term ${\left(P_{d}^{\left(P\right)}\right)}^{G}$ remains close to unity even for large *G*, so

(32) $${\mathrm{FPR}}_{\mathrm{proposed}}\approx P_{d}^{\left(I\right)}\cdot \frac{G}{G}=P_{d}^{\left(I\right)}\approx 0.9976$$

This explains why the proposed scheme’s FPR curve is nearly flat, decreasing only slightly from 0.996 at G=10 to 0.989 at G=60.

Without recovery, however, we have

(33) $${\left(P_{d}^{\left(P\right)}\right)}^{G}={\left(0.9927\right)}^{30}\approx 0.803\;\mathrm{for}\;G=30$$

As *G* increases, the summation saturates at $1/(1-P_{d}^{\left(P\right)})\approx 137$. Thus,

(34) $${\mathrm{FPR}}_{V2V-\mathrm{only}}\to \frac{P_{d}^{\left(I\right)}}{G}\cdot \frac{1}{1-P_{d}^{\left(P\right)}}\propto \frac{1}{G}$$

This inverse relationship explains the steady decline from 0.92 at G=10 to 0.77 at G=60. It shows the importance of trying to maintain the decodability of P-frames as close to unity as possible through the V2N2V retransmissions.

##### *N* (Max Packets per Request)

The parameter *N* affects recovery whenever the receiver cannot determine the exact burst length through gap-based detection. They are long mid-frame bursts (m>2), long end-of-frame bursts (m>2), and all frame-start bursts. We assumed that the burst length follows a geometric distribution with termination probability q=0.656. Consequently, P(m≤N) saturates quickly. By N=3, the probability already reaches 0.959, leaving only 4% of bursts unrecoverable. Additional increases yield marginal gains: N=5 captures 99.5%, and N=10 captures effectively 100%. Moreover, 88% of bursts are short enough for gap-based detection (see Equation (10)), and only 12% require timeout-based recovery where *N* is relevant. Combined with the rapid saturation of P(m≤N), this explains why FPR sensitivity to *N* diminishes quickly.

#### 5.1.6. Discussion on Analytical Model

The sensitivity analysis reveals five key insights about the proposed scheme:

1. Robustness to channel degradation: The proposed scheme maintains high FPR even when the packet loss rate increases significantly, which is valuable for vehicular environments where channel conditions vary rapidly.
2. Parsimonious retransmission suffices: Even with N=3, FPR reaches 0.99, demonstrating that selective packet-level recovery is highly cost-effective.
3. Edge support is essential: The proposed scheme outperforms V2V-only only when $d_{\min}<48.5$ ms, with full recovery requiring $d_{\min}<4$ ms, necessitating MEC@gNB deployment.
4. Coding efficiency is preserved: Unlike V2V-only where FPR ∝1/G, the proposed scheme maintains stable FPR even at G=60, enabling aggressive compression.
5. Timing constraints are coupled: The parameters $T_{\mathrm{SLO}}$, $T_{\mathrm{inter}}$, and $d_{\min}$ are interdependent; e.g., $T_{\mathrm{inter}}>80$ ms or $T_{\mathrm{SLO}}<31$ ms can preclude recovery for certain burst scenarios.

### 5.2. Trace-and-Model-Driven Simulation

**Figure 7:**
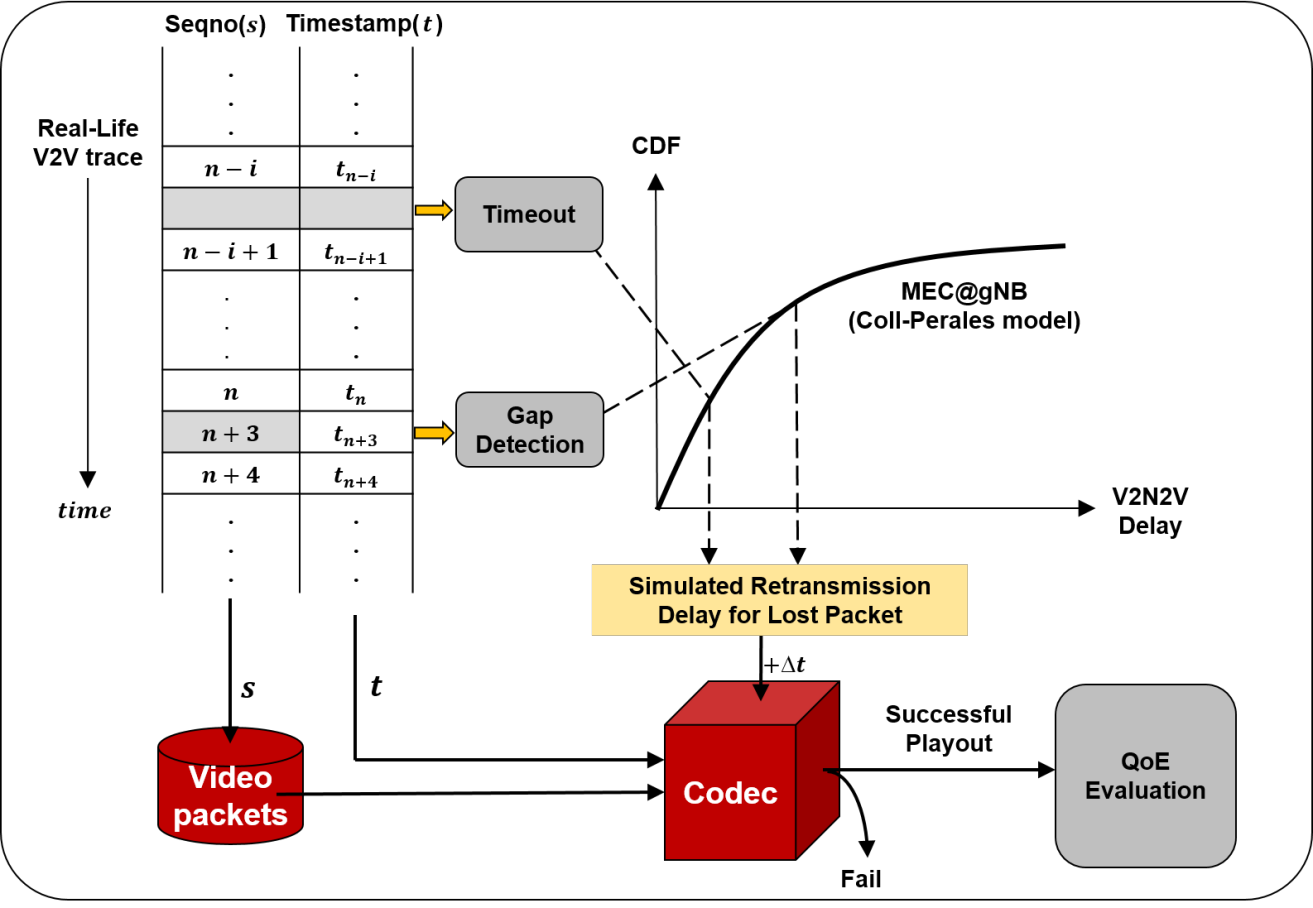
Trace-and-model-driven simulation architecture.

We conducted trace-and-model-driven simulation based on V2V communication traces from Section 3.1, which are collected in actual urban driving environments. Figure 7 shows the simulator architecture. It emulates dynamic V2V packet loss patterns by replaying the collected traces. When a V2V packet loss is detected, the MEC@gNB delay model [3] is consulted to determine the V2N2V latency for retransmission, reflecting a target deployment scenario aligned with current 3GPP standardization for URLLC-oriented V2X services. This hybrid approach taken in this paper is necessitated by the unavailability of MEC-based V2N2V traces.

The simulator applies the logic presented in Section 4.1 to each packet arrival event to determine whether loss was detected by sequence number gap or timeout. When loss is detected, two delay intervals occur in the recovery process via the V2N2V path: (1) $\Delta t_{req}$ required for the retransmission request to reach the sender via the V2N2V path, and (2) $\Delta t_{retx}$ required for the requested packet to be retransmitted and reach the receiver. To reflect this, the simulator sequentially extracts two delay samples from the MEC@gNB delay model and adds them. That is, the final packet arrival time is calculated as

$$t^{\prime }=t+\Delta t_{\mathrm{req}}+\Delta t_{\mathrm{retx}}$$

where we set $t^{\prime }=t$ when there is no loss. Then, the simulator checks whether each packet’s arrival time ($t^{\prime }$) satisfies the SLO (50 ms). When all packets belonging to the same frame are received within the SLO, the resulting frame is input to the codec to attempt decoding. If the decoding is successful for final playback, QoE evaluation is performed.

#### 5.2.1. Compared Strategies

We compared and analyzed the following four transmission strategies:

- V2V Only (Baseline): transmission via a V2V single path only without any retransmission mechanism.
- Gap Only: strategy that detects only discontinuity (gap) in received sequence numbers to request retransmission of lost packets.
- Gap+Timeout (Proposed): the proposed framework using both a gap trigger and timeout trigger.
- PLR Switching [5]: strategy that switches the transmission path based on application SLO (loss rate < 1%). When the V2V path Packet Loss Ratio (PLR) exceeds the threshold, the entire path switches to V2N2V, and it returns to V2V when the V2V path PLR stabilizes for a certain period. It is a hysteresis-based approach, which maintains cellular connectivity for a minimum retention time after switch.

Among cellular-supported multi-RAT approaches with cost consideration, we compare against Brahim et al. [5] as it is the only prior work addressing cost-effective inter-vehicle real-time video streaming. Some approaches target disparate application settings: Internet content download with inter-vehicle cooperation [18,24], periodic beacon transmission [19,20,22,23,25], and V2I-based download scenarios [21]. Others take approaches that are not easily comparable, such as exploiting a location-based QoS map [25] or entirely offloading traffic to cellular upon DSRC congestion [22].

#### 5.2.2. Parameters Configuration

Timeout-based loss detection and retransmission parameters were set based on the statistics from the V2V communication traces.

- Intra-frame timeout ($T_{Intra}$): V2V experiment results showed that the $P_{99}$ of inter-packet arrival intervals within a frame was 2.11 ms. It reflects the 1 ms packet pacing interval with jitter. Considering this, $T_{Intra}=2.5$ ms was used. In real-world deployments, this parameter can be flexibly configured by considering the expected packet inter-arrival time determined by the resource allocation strategy and adding a margin for network jitter, which varies depending on the specific hardware performance and the driving environment.
- Inter-frame timeout ($T_{Inter}$): In a 30 fps video streaming experiment, the $P_{99}$ value of inter-frame packet arrival intervals including the software encoder (e.g., FFmpeg) processing delay and protocol stack delay was 59.08 ms. Accordingly, $T_{Inter}=60$ ms was used. In practical applications, this parameter can be adaptively updated based on the target video frame rate (1/FPS) and the specific processing capabilities of the system in use.
- The maximum number of packets *N* that can be requested in timeout-based retransmission is limited to 3. Under our video encoding configuration (640 × 480, H.264, RTP), a single video frame averages 3.28 packets with a median of two. Since burst losses primarily occur among packets within the same frame rather than across frames, and Figure 3c shows that 95.92% of loss events involve three or fewer consecutive packets, limiting timeout-based retransmission requests to at most three packets effectively covers the majority of loss patterns in practical V2V environments. However, *N* is a design parameter that should be adjusted according to video resolution, encoding scheme, and frame structure to suit specific application requirements.

In the considered MEC@gNB deployment, the mean end-to-end V2N2V latency is 3.22 ms. Consequently, retransmission round trips typically remain within approximately 10 ms, which is well below the 50 ms delay SLO. Thus, retransmission latency itself does not dominate packet reception outcomes; rather, the packet recovery performance depends primarily on when loss is detected. Packet arrival timing may deviate from expectations due to V2V HARQ retransmissions and variability in frame capture and packetization, affecting the time available for recovery within the SLO. The simulation processes over 170,000 packets across more than 45,000 video frames, providing sufficient coverage to reflect these timing-related effects.

#### 5.2.3. Metrics

We evaluate performance using two categories of metrics. The first category consists of network-layer metrics that characterize the packet and frame delivery:

- Packet Reception Ratio (PRR): the ratio of packets successfully received within the SLO to the total packets transmitted.
- Cellular Network Utilization: the fraction of total video traffic transmitted via the cellular path for retransmission. Lower utilization indicates a more efficient use of cellular resources.

The second category consists of application-layer QoE metrics that reflect the end-user viewing experience:

- Frame Playback Ratio (FPR): the ratio of frames successfully decoded and displayed to the total frames transmitted, accounting for codec dependencies where the loss of a reference frame renders dependent frames undecodable.
- Stall Count: the number of playback interruptions caused by missing frames at their scheduled display time.
- Structural Similarity Index (SSIM): a perceptual quality metric quantifying the visual similarity between transmitted and received video frames, ranging from 0 (no similarity) to 1 (identical).

### 5.3. Simulation Results

#### 5.3.1. PRR

This section evaluates PRR based on 5GAA’s V2V video streaming use case requirements (end-to-end latency < 50 ms) [2].

##### Overall Reliability

**Figure 8:**
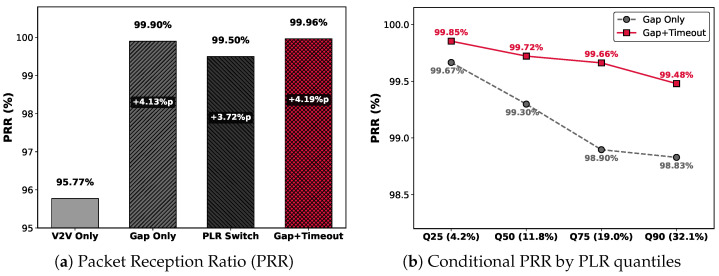
Transmission reliability analysis comparison: (**a**) PRR of each strategy, and (**b**) conditional PRR across V2V PLR quantiles.

Figure 8a shows the PRR for the entire trace. V2V Only recorded 95.77% PRR, failing to meet target application requirements (99%). PLR Switching achieved a high PRR of 99.50%, but it has limitations of excessive cellular data consumption, as will be shown in the subsequent cost analysis. In contrast, Gap+Timeout achieved 99.96% PRR, perfectly compensating for V2V reliability limitations with V2N2V to demonstrate nearly lossless performance.

##### Robustness in Deep Fading Conditions

The difference between Gap Only (99.90%) and Gap+Timeout (99.96%) may appear negligible in PRR numbers. However, an in-depth analysis by channel state clearly reveals the robustness difference of the proposed framework. To precisely analyze the V2V channel loss rate variability, we segmented the entire trace into PLR intervals using the Binary Segmentation algorithm [28] and analyzed the conditional PRR for each interval, and the results are shown in Figure 8b. Analysis results show that in severe channel degradation intervals (Q75, top 25% high-loss interval) where the V2V PLR exceeds 19%, Gap Only’s PRR drops to 98.90%, failing to achieve SLO (99%). This is because Gap Only depends on subsequent packet arrivals and cannot quickly recover losses occurring in consecutive packet loss (burst loss) or traffic tail loss. In contrast, Gap+Timeout maintained a stable reliability of 99.66% even under such adverse conditions through the proactive timeout mechanism operation. Particularly in the worst communication environment Q90 (PLR ≥ 32.1%) interval, it recorded 99.49% PRR, demonstrating its robustness.

#### 5.3.2. Resource Efficiency and Cost

##### Cellular Network Utilization

**Figure 9:**
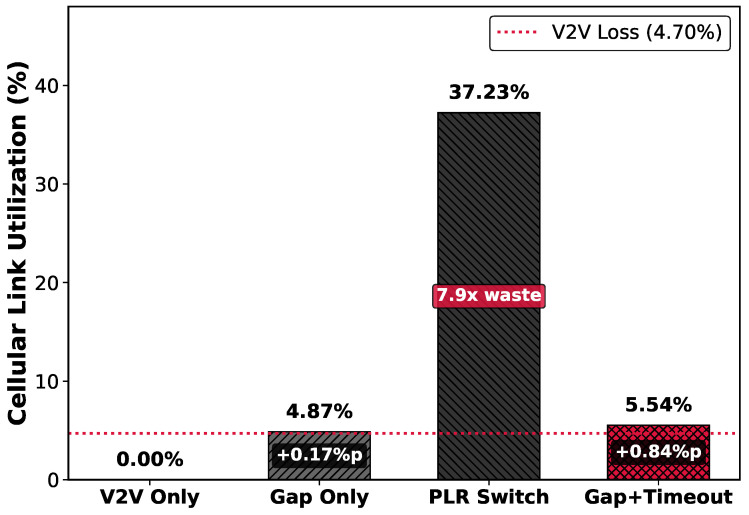
Cellular link utilization comparison, demonstrating the efficiency of the proposed framework.

Pure data loss on the V2V path measured in the experimental interval is 4.70% of total traffic. This corresponds to the theoretical lower bound of data that must be invested for data integrity. In Figure 9, Gap Only recorded 4.87% cellular link utilization. This is close to the theoretical lower bound even including the retransmission request overhead, demonstrating its lean operation. In contrast, PLR Switching recorded 37.23%, wasting 7.9 times the actual required amount (4.70%). Compared to this, Gap+Timeout recorded 5.54% utilization. This is only a 0.84% increase over the theoretical minimum, demonstrating maximized resource efficiency while achieving lossless-level reliability (99.96% PRR).

##### Monetary Cost

To quantify the economic value of data efficiency, we assume a vehicle operating 2 h daily for commuting with the above utilization efficiency. Considering a typical high-definition video streaming bitrate of approximately 4 Mbps, continuously serving video to surrounding vehicles for 2 h produces approximately 3.8 GB of data. Of this, Gap Only must transmit 177 MB, Gap+Timeout 209 MB, and PLR Switching 1.4 GB via the cellular path. When consuming data at this rate for approximately 20 days per month, these three strategies transmit 3.5 GB, 4.2 GB, and 28 GB, respectively, via the cellular path. If the 5G sidelink wireless environment is much worse than that of downtown Seoul measured in our study, the cellular path usage would exceed the data amounts presented above, making economic considerations non-negligible for connected vehicles.

The data consumption difference (e.g., 4.2 GB vs. 28 GB monthly) has varying economic impact depending on data plans:

- Unlimited plans with throttling (assume 22 GB threshold used by AT&T): Gap+Timeout stays below threshold; PLR Switching triggers rate limiting, affecting concurrent services.
- Tiered plans (e.g., 10/20/30 GB tiers): Gap+Timeout remains in lower tiers; PLR Switching forces tier upgrades ( $20–40/month).
- Pay-per-GB plans (e.g., $10/GB overage): direct savings of $240/month per vehicle.

Thus, these economic considerations could be decisive in whether vehicle owners allow their cellular connectivity to support V2X, ultimately shaping whether the promising applications arising from the integration of V2N and V2X technologies will become a reality. Beyond monetary cost, reducing cellular utilization benefits network operators by enabling a higher vehicle density per cell, which is increasingly critical as hybrid transmission becomes widespread.

**Figure 10:**
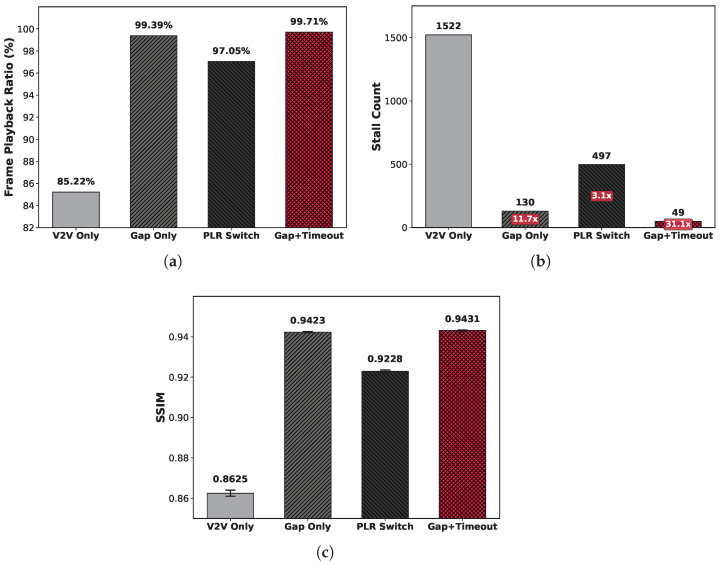
Comparison of QoE metrics: (**a**) Frame Playback Ratio (FPR); (**b**) stall count (during 26 minutes of live vehicle-to-vehicle video streaming simulation); (**c**) average SSIM.

While the preceding analysis focused on network-level performance, we also evaluated user-perceived quality through QoE metrics: the Frame Playback Ratio (FPR), stall count, and video quality (SSIM). Figure 10 shows the QoE performance comparison results for each transmission strategy.

#### 5.3.3. Frame Playback Ratio

Due to the packet-to-frame dependency and the structural dependency of the video frames discussed in Section 3.2, packet loss is amplified nonlinearly in FPR. Figure 10a shows the FPR for each strategy. As established in Section 3.2, the V2V Only results clearly reveal single-path reliability limitations. The 85.22% FPR corresponds to four to five frames being continuously lost per second for 30 fps video, causing video discontinuity that could impede driver situation awareness and pose a safety threat.

Hybrid strategies significantly improved performance, but gaps exist among them. PLR Switching recorded 99.50% PRR but only 97.05% FPR, while the proposed Gap+Timeout achieved 99.71% FPR from 99.96% PRR. Notably, a mere 0.46% difference in PRR expanded to 2.66% in FPR (over 5× amplification), demonstrating that minor network-level reliability differences can result in substantial application-level impact. Consequently, the proposed framework saves approximately 50 additional frames per minute compared to PLR Switching, providing drivers with seamless visual information during high-speed driving.

#### 5.3.4. Stall Count

Playback stalls impair user immersion and disrupt information delivery. Figure 10b shows the total stall count during 26 min of simulation experiments. V2V Only recorded 1522 stalls as reported in Section 3.2. In contrast, PLR Switching generated 497 stalls, while the proposed Gap+Timeout reduced this to 49, which is a 10× reduction from that of PLR Switching. Due to the high inter-packet dependency in video data, even minor network-level losses can cause stalls. These results indicate that our proposed framework, by immediately recovering lost data through the reliability-guaranteed V2N2V path, is effective in maintaining video streaming QoE.

#### 5.3.5. Video Quality

Figure 10c shows the average SSIM. V2V Only recorded 0.8625 as reported in Section 3.2, which is substantially lower than the hybrid strategies, indicating that drivers would clearly perceive a significant degradation in video quality. Among hybrid strategies, the proposed Gap+Timeout achieved the highest SSIM of 0.9431, slightly exceeding Gap Only (0.9423) and notably outperforming PLR Switching (0.9228). Flynn et al. [29] reported that the Just Noticeable Difference (JND) threshold for SSIM is approximately 0.95, below which human observers begin to perceive quality degradation. While PLR Switching falls well below this threshold, Gap+Timeout approaches it closely, suggesting that modest further improvements could achieve perceptually lossless quality. This result demonstrates that the proposed framework minimizes error propagation from packet loss and provides quality closest to the original video.

### 5.4. Model Validation

We validate the analytical model developed in Section 5.1 by comparing its predictions against the simulation results presented above.

#### 5.4.1. Parameter Fitting

The burstiness factor β is determined by fitting the V2V-only FPR model to match the simulation result:

(35) $$\beta^{*}=\arg\min_{\beta }{\left({\mathrm{FPR}}_{\mathrm{model}}\left(\beta \right)-{\mathrm{FPR}}_{\mathrm{sim}}^{V2V}\right)}^{2}$$

With ${\mathrm{FPR}}_{\mathrm{sim}}^{V2V}=0.8522$ from simulation, fitting yields $\beta^{*}=0.063\ll 1$, indicating highly bursty error patterns.

#### 5.4.2. Numerical Evaluation

Using the fitted β=0.063 with baseline parameters:

- Frame failure: $P_{f}^{\left(I\right)}=0.0527$, $P_{f}^{\left(P\right)}=0.0071$;
- Recovery (I-frame): $P_{\mathrm{rec}}^{\left(I\right)}\approx 0.9487$ (limited by tight $B_{\mathrm{timeout}}^{\left(I\right)}$ and m>N cases);
- Recovery (P-frame): $P_{\mathrm{rec}}^{\left(P\right)}\approx 0.9891$ (better due to smaller frame size and larger $B_{\mathrm{timeout}}^{\left(P\right)}$).

Substituting into Equations (28) and (29), the frame decode probabilities become

(36) $$\begin{array}{cc} P_{d}^{\left(I\right)} & =0.9473+0.0527\times 0.9487=0.9973 \end{array}$$

(37) $$\begin{array}{cc} P_{d}^{\left(P\right)} & =0.9929+0.0071\times 0.9891=0.9999 \end{array}$$

Substituting into Equation (8) yields ${\mathrm{FPR}}_{\mathrm{prop}}\approx 0.9973$.

#### 5.4.3. Validation Results

Table 6 compares the model predictions with the simulation results.

The V2V-only results match exactly by construction: *p* is measured from the trace and β is fitted to FPR. The meaningful validation is whether these parameters predict the proposed scheme’s performance.

For the proposed scheme:

- PRR: Model predicts 0.9999 vs. simulation 0.9996 (error: +0.03%);
- FPR: Model predicts 0.9973 vs. simulation 0.9971 (error: +0.02%).

Both metrics are slightly overestimated, indicating that the model is optimistic about recovery success. This bias arises from simplifying assumptions: a uniform distribution of burst start positions, independence between consecutive bursts, and perfect V2N2V reliability.

Despite this small prediction error, the model correctly captures the key qualitative insight: V2N2V backup recovery transforms moderate V2V reliability (PRR 95.77%, FPR 85.22%) into near-perfect performance (PRR 99.96%, FPR 99.71%).

We acknowledge that the validation is limited by having only one simulation scenario. A more rigorous validation would require multiple channel conditions, testing whether the fitted parameters (β,q) generalize. We leave this extension for future work.

### 5.5. Qualitative Analysis on Scalability

While our evaluation focuses on a single vehicle pair, the architectural characteristics of the proposed framework suggest robust scalability in multi-vehicle scenarios.

In high-density scenarios, the Sidelink Channel Busy Ratio (CBR) increases, leading to higher packet loss. Conventional strategies, such as PLR Switching, typically offload the entire video stream to the cellular network when loss exceeds a threshold. This behavior can cause a sudden surge in cellular bandwidth demand. In contrast, by design, our framework retransmits only the lost packets. Therefore, even as sidelink congestion worsens, the cellular traffic load generated by our method increases only in proportion to the loss rate rather than spiking to 100%. This structural efficiency is expected to significantly reduce the aggregate load on the cellular network compared to full-offloading schemes.

Regarding the V2N2V path, multi-vehicle traffic may increase the processing load on the MEC server. However, the MEC@gNB architecture fundamentally reduces network latency by eliminating the need for traffic to traverse the core network and the internet. This architectural advantage provides significant latency headroom compared to centralized routing. Even if queuing delays occur due to high server load, this inherent margin allows the system to absorb the delays while remaining within the 50 ms SLO.

## 6. Conclusions

This paper addressed the challenge of achieving reliable and cost-effective real-time video streaming between connected vehicles equipped with both V2V sidelink and cellular (V2N2V) interfaces. Through measurement studies in Seoul urban driving environments, we characterized the complementary nature of these paths: V2V provides ultra-low latency (mean 2.99 ms) but imperfect reliability (95.77% PRR), while V2N2V achieves perfect reliability but suffers from high latency variability that can violate stringent V2X timing requirements. We proposed a hybrid framework that uses V2V as the primary path while selectively utilizing V2N2V only for retransmitting lost packets. The key innovation is a dual loss detection mechanism combining gap-based and timeout-based triggers that leverage RTP header information. The gap-based trigger provides immediate detection upon sequence number discontinuity, while the timeout-based trigger handles burst and tail losses using differentiated intra-frame (2.5 ms) and inter-frame (60 ms) timeouts. Trace-driven simulation demonstrated the effectiveness of the proposed approach. Gap+Timeout achieved 99.96% PRR and 99.71% FPR, reduced stalls by 10× compared to PLR Switching (49 vs. 497), and improved video quality (SSIM: 0.9431 vs. 0.9228). Most importantly, the framework maintained cellular utilization at only 5.54% (merely 0.84%p above the theoretical lower bound) while PLR Switching consumed 37.23%, representing approximately a 7× cost reduction. Minimizing cellular usage is critical for bridging V2N and V2X technologies, as economic pressures may ultimately determine whether vehicle owners permit their cellular connectivity to support V2X services. By demonstrating that reliable video streaming is achievable with minimal cellular cost, this work contributes toward enabling the promising applications that arise from integrating connected vehicles with V2X capabilities.

## Figures and Tables

**Table 1:** 5GAA-defined real-time video-based V2X use cases (SLO: 50 ms latency, 99% reliability) [2].

Use Case	Description
See-Through for Passing	Streaming real-time video from a leading vehicle to a host vehicle to visualize the road ahead, specifically to support safe passing maneuvers.
Obstructed View Assist	Providing alternative views from infrastructure (CCTV) or other vehicles to resolve blind spots caused by obstacles, typically at intersections or driveways.
Non-analysed Sensor Signal Sharing	Sharing raw sensor data streams (e.g., LiDAR, Camera) instead of processed object lists. Used to enhance the host vehicle’s sensor fusion and perception reliability.

**Table 2:** Comparison of Multi-RAT V2X communication strategies.

Prior Work	Assumed RATs	Multi-RAT Usage	Cellular Use Minimization	Cellular Usage Logic	Target Usecase
Sepulcre [10]	DSRC, WiFi, TVWS	Select	N/A (No Uu)	N/A	Cooperative Perception
Jacob [11]	DSRC, PC5	Duplicate	N/A (No Uu)	N/A	Platooning
Yacheur [13,14]	DSRC, PC5	Select, Duplicate, Split	N/A (No Uu)	N/A	Platooning, See-through
Jacob [15]	DSRC, PC5, Uu	Select, Duplicate, Split	No	Use cellular (Uu) when selected communication profile requires Uu either alone or in combination with V2V	Connected Autonomous Driving
Albonda [16]	PC5, Uu	Select	No	Select Uu when uplink/downlink SINR exceeds the sidelink SINR if sufficient resource is available	Safety Messaging
González [17]	PC5, Uu	Select, Duplicate	No	Use Uu for centralized sidelink resource scheduling and V2N services or when redundantly transmitting data alongside PC5	Advanced V2X Services
Brahim [5]	DSRC, Uu	Select	Yes	Use Uu only when DSRC PLR crosses threshold; revert after *n* good windows	Real-time V2V video
Hui [18]	DSRC, Uu	Select	Yes	Select Uu if the cellular link results in a lower total cost than V2I/V2V communication	On-Demand Content Downloading
Mir [19,20]	DSRC, Uu	Select	Yes	Switch to Uu only when DSRC congestion persists despite rate control allowed by QoS requirements	Periodic Safety Messaging
Altahrawi [21]	DSRC, WiFi, Uu	Select	Yes	Use Uu only to maintain service continuity when RSU-based DSRC/WiFi links are unavailable or insufficient to meet QoS requirements	V2I Download
Mir [22]	DSRC, Uu	Select	Yes	Offload to Uu only when DSRC becomes congested while the cellular network has sufficient capacity	Periodic Safety Messaging
Khalid [23]	DSRC, PC5, Uu	Select, Duplicate	Yes	Use Uu if DSRC/PC5 cannot satisfy communication requirements	Periodic Safety Messaging
Chowdhury [24]	WiFi, DSRC, Uu	Select	Yes	Use WiFi/DSRC first; cellular only if no alternative source can deliver content	CDN-Based Live Video Streaming
Bréhon [25]	DSRC, PC5, Uu	Select	Yes	Select Uu as a fallback when DSRC/PC5 fail to meet predefined latency bound	Periodic Safety Messaging

(1) RATs: DSRC = IEEE 802.11p; PC5 = 3GPP sidelink interface; Uu = 3GPP cellular uplink/downlink interface; TVWS = TV white space spectrum. (2) Multi-RAT Usage: Select = using only a single RAT at a time; Duplicate = transmitting identical packets over multiple RATs; Split = distributing data streams or packets across different RATs. (3) Cellular Use Minimization: N/A = no cellular path involved; No = cellular is used without cellular usage minimization; Yes = implicit or explicit cellular usage minimization or cost optimization.

**Table 3:** NR Sidelink Mode 2 configuration parameters.

Parameter	Value
Center Frequency	5875 MHz
Bandwidth	20 MHz
Subcarrier Spacing (SCS)	15 kHz (μ=0)
Subchannel Size	15 RBs
Number of Subchannels	7
Resource Reserve Period (RRP)	0 (Dynamic resource selection)
MCS Table/Index	64QAM/26
Max HARQ Transmissions	4
PSFCH Period	1 slot
Tx Power	20 dBm

**Table 4:** V2N2V path latency: measured vs. standard architectures.

Architecture	Mean	$P_{90}$	$P_{99}$	Meets 50 ms, 99%
Our Measurement (Multi-MNO/Internet)	39.40 ms	59.86 ms	120.33 ms	No
Coll-Perales [3] (Centralized)	∼17 ms	∼58.06 ms	∼152.44 ms	No
Coll-Perales [3] (MEC@gNB)	∼3.22 ms	∼3.595 ms	∼7.473 ms	Yes
3GPP URLLC Target	<30 ms	<45 ms	<50 ms	Yes

**Table 5:** Base parameter set.

Symbol	Description	Value
Video Parameters		
$N_{f}^{\left(I\right)}$	I-frame size	20.41 packets
$N_{f}^{\left(P\right)}$	P-frame size	2.69 packets
*G*	GoP size	30 frames
$T_{\mathrm{frame}}$	Frame interval	33.33 ms
Channel Parameters		
*p*	Packet loss rate	0.0423
β	Burstiness factor	(fitted)
*q*	Burst termination rate	0.656
Timing Parameters		
$T_{\mathrm{SLO}}$	Latency deadline	50 ms
$T_{\mathrm{inter}}$	Inter-frame timeout	60 ms
$T_{\mathrm{intra}}$	Intra-frame timeout	2.5 ms
δ	Packet pacing interval	1.0 ms
Recovery Parameters		
*N*	Max packets per request	3
$d_{\min}$	Min V2N2V delay	2.5 ms
μ	Log-normal location	−0.65
σ	Log-normal scale	0.68

**Table 6:** Model validation results.

	V2V-Only		Proposed	
Metric	Model	Simulation	Model	Simulation
PRR	0.9577	0.9577	0.9999	0.9996
FPR	0.8522 (fitted)	0.8522	0.9973	0.9971

## Data Availability

Data available on request due to restrictions.

## References

[1] U.S. Department of Transportation, Intelligent Transportation Systems Joint Program Office (2024). Vehicle-to-Everything (V2X) Technology Executive Briefing.

[2] 5G Automotive Association (5GAA) (2025). C-V2X Use Cases and Service Level Requirements (Vols. I, II and III).

[3] Coll-Perales B., Lucas-Estañ M.C., Shimizu T., Gozalvez J., Higuchi T., Avedisov S., Altintas O., Sepulcre M. (2022). End-to-end V2X latency modeling and analysis in 5G networks. IEEE Trans. Veh. Technol..

[4] 3GPP (2022). 5G System Enhancements for Edge Computing; Stage 2 (Release 17).

[5] Brahim M.B., Mir Z.H., Znaidi W., Filali F., Hamdi N. (2017). QoS-aware video transmission over hybrid wireless network for connected vehicles. IEEE Access.

[6] Zhang X., Li J., Zhou J., Zhang S., Wang J., Yuan Y., Liu J., Li J. (2025). Vehicle-to-Everything Communication in Intelligent Connected Vehicles: A Survey and Taxonomy. Automot. Innov..

[7] Naeem M.A., Chaudhary S., Meng Y. (2024). Road to efficiency: V2v enabled intelligent transportation system. Electronics.

[8] Naseh D., Shinde S.S., Tarchi D. Enabling intelligent vehicular networks through distributed learning in the non-terrestrial networks 6G vision. Proceedings of the 28th European Wireless Conference on European Wireless 2023, VDE.

[9] Naseh D., Bozorgchenani A., Tarchi D. (2025). Deep Reinforcement Learning for Edge-DASH-Based Dynamic Video Streaming. Proceedings of the 2025 IEEE Wireless Communications and Networking Conference (WCNC).

[10] Sepulcre M., Gozalvez J. (2019). Heterogeneous V2V communications in multi-link and multi-RAT vehicular networks. IEEE Trans. Mob. Comput..

[11] Jacob R., Anwar W., Fettweis G., Pohlmann J. (2019). Exploiting multi-RAT diversity in vehicular ad-hoc networks to improve reliability of cooperative automated driving applications. Proceedings of the 2019 IEEE 90th Vehicular Technology Conference (VTC2019-Fall).

[12] (2010). IEEE Standard for Information Technology–Local and Metropolitan Area Networks–Specific Requirements–Part 11: Wireless LAN Medium Access Control (MAC) and Physical Layer (PHY) Specifications Amendment 6: Wireless Access in Vehicular Environments.

[13] Yacheur B.Y., Ahmed T., Mosbah M. (2023). DRL-based RAT selection in a hybrid vehicular communication network. Proceedings of the 2023 IEEE 97th Vehicular Technology Conference (VTC2023-Spring).

[14] Yacheur B.Y., Ahmed T., Mosbah M. (2023). Efficient DRL-based selection strategy in hybrid vehicular networks. IEEE Trans. Netw. Serv. Manag..

[15] Jacob R., Franchi N., Fettweis G. (2018). Hybrid V2X communications: Multi-RAT as enabler for connected autonomous driving. Proceedings of the 2018 IEEE 29th Annual International Symposium on Personal, Indoor and Mobile Radio Communications (PIMRC).

[16] Albonda H.D.R., Pérez-Romero J. (2020). A new mode selection and resource reuse strategy for V2X in future cellular networks. Proceedings of the 2020 IEEE 91st Vehicular Technology Conference (VTC2020-Spring).

[17] González E.E., Garcia-Roger D., Monserrat J.F. (2022). LTE/NR V2X communication modes and future requirements of intelligent transportation systems based on MR-DC architectures. Sustainability.

[18] Hui Y., Su Z., Luan T.H., Cai J. (2019). A Game Theoretic Scheme for Optimal Access Control in Heterogeneous Vehicular Networks. IEEE Trans. Intell. Transp. Syst..

[19] Mir Z.H., Toutouh J., Filali F., Alba E. (2015). QoS-aware radio access technology (RAT) selection in hybrid vehicular networks. Proceedings of the International Workshop on Communication Technologies for Vehicles.

[20] Mir Z.H., Toutouh J., Filali F., Ko Y.B. (2020). Enabling DSRC and C-V2X integrated hybrid vehicular networks: Architecture and protocol. IEEE Access.

[21] Altahrawi M., Abdullah N.F., Nordin R. (2022). Service-oriented LSTM multi-criteria RAT selection scheme for vehicle-to-infrastructure communication. IEEE Access.

[22] Mir Z.H., Dreyer N., Kürner T., Filali F. (2024). Investigation on cellular LTE C-V2X network serving vehicular data traffic in realistic urban scenarios. Future Gener. Comput. Syst..

[23] Khalid I., Maglogiannis V., Naudts D., Shahid A., Moerman I. (2024). Optimizing hybrid v2x communication: An intelligent technology selection algorithm using 5g, c-v2x PC5 and dsrc. Future Internet.

[24] Chowdhury D.R., Nandi S., Goswami D. (2024). Cost-effective live video streaming for internet of connected vehicles using heterogeneous networks. Ad Hoc Netw..

[25] Bréhon-Grataloup L., Kacimi R., Beylot A.-L. (2025). Reliable multi-RAT connectivity in urban V2X architectures: An experimental campaign. Ad Hoc Netw..

[26] Zanforlin M., Munaretto D., Zanella A., Zorzi M. (2014). SSIM-based video admission control and resource allocation algorithms. Proceedings of the 2014 12th International Symposium on Modeling and Optimization in Mobile, Ad Hoc, and Wireless Networks (WiOpt).

[27] (2020). Intelligent Transport Systems—Station and Communication Architecture.

[28] Truong C., Oudre L., Vayatis N. (2020). Selective review of offline change point detection methods. Signal Process..

[29] Flynn J.R., Ward S., Abich J., Poole D. (2013). Image Quality Assessment Using the SSIM and the Just Noticeable Difference Paradigm. Proceedings of the Engineering Psychology and Cognitive Ergonomics. Understanding Human Cognition (EPCE 2013).

